# Effects of Beta-Alanine on Muscle Carnosine and Exercise Performance:A Review of the Current Literature

**DOI:** 10.3390/nu2010075

**Published:** 2010-01-25

**Authors:** Julie Y. Culbertson, Richard B. Kreider, Mike Greenwood, Matthew Cooke

**Affiliations:** 1 Exercise and Sport Nutrition Laboratory, Department of Health and Kinesiology, Texas A&amp;M University, College Station, TX 77845, USA; Email: RKreider@hlkn.tamu.edu; 2 Department of Health, Human Performance and Recreation, Baylor University, Waco, TX 73019, USA; Email: mikegreenwood18@gmail.com; 3 Schools of Medicine &amp; Health Movement Studies, The University of Queensland, Herston, Queensland, Australia; Email: m.cooke@uq.edu.au

**Keywords:** creatine monohydrate, anaerobic capacity, muscular fatigue, ergogenic aids

## Abstract

Muscle carnosine has been reported to serve as a physiological buffer, possess antioxidant properties, influence enzyme regulation, and affect sarcoplasmic reticulum calcium regulation. Beta-alanine (β-ALA) is a non-essential amino acid. β-ALA supplementation (e.g., 2-6 grams/day) has been shown to increase carnosine concentrations in skeletal muscle by 20-80%. Several studies have reported that β-ALA supplementation can increase high-intensity intermittent exercise performance and/or training adaptations. Although the specific mechanism remains to be determined, the ergogenicity of β-ALA has been most commonly attributed to an increased muscle buffering capacity. More recently, researchers have investigated the effects of co-ingesting β-ALA with creatine monohydrate to determine whether there may be synergistic and/or additive benefits. This paper overviews the theoretical rationale and potential ergogenic value of β-ALA supplementation with or without creatine as well as provides future research recommendations.

## 1. Introduction

During moderate to high-intensity exercise, hydrogen ions (H+) begin to accumulate leading to a drop in intramuscular pH and ultimately influencing muscle performance [1]. The greater the reliance on glycolysis as the primary energy system (as seen with high-intensity exercise), the greater production of lactic acid and H+, thus leading to further decreases in intramuscular pH. This decrease in intramuscular pH has been suggested to be linked to fatigue-induced increases in muscle activation and electromyographic (EMG) amplitude [2,3]. Thus, if the intramuscular pH decline can be prevented or delayed, the fatigue induced EMG increase may also be delayed [4]. Beta-alanine (β-ALA) supplementation has been shown to increase muscle carnosine levels, which can act as a buffer to reduce the acidity in the active muscles during high-intensity exercise [5,6,7]. β-ALA supplementation has been shown to have beneficial effects on exercise performance variables such as cycling capacity [6], ventilatory threshold, and time to exhaustion [8]. For this reason β-ALA has become a widely used nutritional supplement for improving high-intensity exercise performance [4,5,6,9,10]. Creatine monohydrate supplementation has also been shown to have ergogenic effects by increasing the availability of phosphocreatine (PCr), total creatine concentrations in the muscle, high intensity exercise performance, and training adaptations [11]. For this reason, several studies have assessed whether co-ingesting β-ALA with creatine may have synergist and/or additive effects on exercise capacity and/or training adaptations [4,10,12]. The purpose of this article is to review the theoretical rationale and available scientific evidence assessing the potential ergogenic value of supplementing the diet with β-ALA with or without creatine. In addition, to discuss areas that future research should address. This was accomplished by conducting a thorough review of the published literature related to the physiological effects of carnosine and the role of β-ALA and creatine supplementation on carnosine levels, creatine levels, and exercise performance. 

## 2. Carnosine

**Figure 1:**
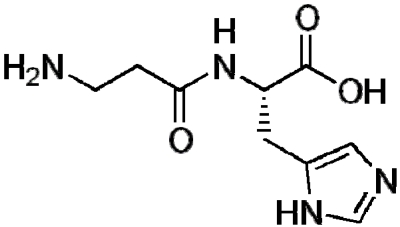
Chemical structure of carnosine.

Carnosine (β-alanyl-L-histidine) is a naturally-occurring histidine-containing compound found in many animal tissues, including skeletal muscle, which is the most abundant source. Carnosine is a multifunctional dipeptide with many roles including buffering [13,14], fighting free radicals [15,16], enzyme regulation [17] and sarcoplasmic reticulum calcium (Ca^2+^) regulation [18,19]. Carnosine is broken down in the body by carnosinase, which is found in most tissues except skeletal muscle, partially explaining why carnosine concentrations are highest in this tissue [19]. Figure 1 shows the chemical structure of carnosine. 

Carnosine in human skeletal muscle generally ranges between 5-10 mM wet weight or 15-40 mmol/kg dry weight [5]. Concentrations differ among animal species, in part due to the differences in muscle mass [20]. For example, horses have been reported to have higher carnosine concentrations than greyhound dogs [21]. Carnosine levels are typically higher in fast-twitch muscle fibers compared to slow-twitch, which corresponds to the observation that animals exposed to frequent sprints, explosive flight behaviors and prolonged hypoxic dives have higher initial carnosine concentrations [5,21,22]. Human athletes involved in anaerobic sports such as sprinters [23,24] and bodybuilders [25] have also been found to have higher intramuscular concentrations of carnosine. Exercise training has been reported to increase resting muscle carnosine concentrations in these athlete types. For example, Gardner and colleagues [26] reported that exercise training increased the plasma carnosinase activity and decreased carnosine excretion leading to greater muscle carnosine concentrations [26]. Moreover, Suzuki and colleagues [27] examined the effects of sprint training on muscle carnosine concentrations. Six male subjects performed sprint training twice a week for a total of 16 training sessions. Each session involved either single (for weeks one and two) or a double (for weeks three through eight) bout of 30 seconds of maximal sprinting on a cycle ergometer with 20 minutes of rest between sprints on the double bout days. Muscle samples were collected from the vastus lateralis one week before training and again two days following the training protocol. Results revealed that muscle carnosine content and mean power output significantly increased after the eight weeks of training [27]. Tallon and coworkers [25] suggested the greater muscle carnosine content in bodybuilders may be due to the chronic exposure to lower pH environments due to their training, differences in their diet such as increased protein intake where carnosine can be found, supplementation use, and/or possible anabolic androgenic steroid use [25].

Carnosine was first discovered as an intracellular pH buffer in 1953 by Severin and colleagues [28] using frog muscle tissue. Subsequent studies examining this relationship in human muscle tissue followed thereafter [13,14,29,30,31,32]. When skeletal muscles are involved in moderate to intense exercise, there is typically a generation of lactic acid and subsequent dissociation into lactate and H+, which can alter the pH levels. It had previously been reported that the majority of protons produced during exercise in the blood were buffered by the bicarbonate buffering system [33]. The pKa of this system is 6.1, which is less than that of carnosine (pKa of 6.83), and thus a greater pH change is needed to elicit benefits from this system. Since the pKa of carnosine is closer to the physiological pH, it is likely that this is utilized sooner as a buffer during high-intensity exercise [1]. The imidazole group on the histidine containing molecules, such as carnosine, makes it especially effective as a buffer. This group has a pKa value close to that of the intracellular pH, therefore one of the nitrogens from the imidazole ring can be used to accept a proton [34]. 

Early studies examined the role of carnosine in animal models. One study, utilizing chromatography methodology to analyze rabbit and pigeon muscle samples, reported muscle dipeptides (mainly carnosine and anserine) accounted for approximately 40% of the pH buffering capability in skeletal muscle [13]. Later, Bump and colleagues [35] examined the carnosine concentrations in different breeds of horses. They compared quarter horses (QH), thoroughbreds (TB) and standardbreds (SB) in order to correlate buffering capabilities of the muscle to fiber type composition. The QH demonstrated less slow-twitch muscle fibers, greater fast-twitch glycolytic fibers, and fewer fast-twitch oxidative muscle fibers compared to the other horses. Results showed QH had significantly greater amounts of carnosine in their muscle. The researchers reported a positive correlation between carnosine concentrations and fast-twitch glycolytic fibers and a negative correlation between carnosine and fast-twitch oxidative fibers. The investigators inferred that intramuscular carnosine acted as an intracellular buffer, although this was not directly measured. A later study conducted by Sewell and associates [36] specifically examined the buffering capability of carnosine in different fiber types of horses. These researchers found that carnosine contributed about 20% of the buffering in type I fibers and up to 46% in Type IIb fibers. These findings are consistent with the findings that less lactic acid is accumulated in Type I fibers due to the lower intensity muscle activity involved with this fiber type. 

An early study by Kraemer and associates of humans utilizing carnosine supplementation [37] reported no effect on the acid-base status or exercise performance using four subsequent 30 second Wingate tests with only two minutes of rest between exercise bouts. In this regard, the researchers evaluated ten trained and ten untrained males who consumed a total of 15 capsules of a supplement containing 1000 mg dibasic sodium phosphate, 204 mg potassium bicarbonate and 12.5 mg L-carnosine over a 3.5 day period. Placebo capsules were matched in sodium and potassium content. Blood samples were taken at baseline prior to any exercise, immediately after each Wingate test, and at three minutes after all exercise was completed. Though intramuscular carnosine levels were not measured, the authors suggested that the amount of carnosine provided to subjects (about 185 mg) may have been too low to have an impact on intramuscular carnosine levels [26,38,39] particularly since previous animal studies had shown increases following a daily dose between 50-200 mg/kg of body weight [38,40]. 

Human studies have shown that lowered pH levels can also negatively affect the excitation-contraction coupling in the skeletal muscle [38,41]. The buffer efficacy in human muscle was examined by calculating the buffering ability over the physiological pH range of 7.1-6.5. This study involved 50 healthy active individuals who underwent a muscle biopsy from the lateral portion of the quadriceps femoris muscle. Anserine and carnosine were analyzed in neutralized perchloric acid extracts using high performance liquid chromatography (HPLC) methods. The Henderson-Hasselbach equation was then used to indirectly calculate the buffer contribution across the pH range of 7.1 to 6.5. It was estimated that carnosine was able to buffer between 2.4 and 10.1 mmol H^+^∙kg^-1^ dry mass, which corresponded to about 7% of the total muscle buffering [9]. Therefore, these results indicated that carnosine played a minimal role in buffering pH. 

Suzuki and coworkers [42] examined the effects of the non-bicarbonate buffers carnosine and anserine. They had eight active males supplement with either a placebo or chicken breast extract (CBEX) soup that contained 1.5 g carnosine and anserine. Subjects then performed ten sets of five second maximal cycle sprints at 7.5% of their body weight as resistance. Blood samples were collected at rest, one minute before exercise, after each exercise set, and immediately after the intervals to measure blood-gas parameters, blood lactate and concentrations of carnosine and anserine. The researchers found that supplementing the diet with CBEX delayed the decrease in bicarbonate during intense exercise, but did not improve performance. These results support the initial use of carnosine as a buffer instead of the bicarbonate system [42]. 

Early studies with carnosine supplementation noted plasma carnosine levels failed to elevate due to the high activity of carnosinase [26]. The researchers were able to measure only 14% of the ingested carnosine in urine suggesting this was due to absorption in the gastrointestinal tract [26]. Later, research pointed towards supplementing with β-ALA and L-histidine instead to raise carnosine levels since these are the precursors to carnosine. Dunnett and Harris [14] discovered that β-ALA was able to increase carnosine in muscle tissue. In their study, they supplemented horses with both β-ALA and L-histidine and found β-ALA to have an additive response suggested to be due to the increase β-amino acid transport across the gastrointestinal tract. This was not observed for L-histidine, thus speaking to the efficacy of β-ALA instead to increase carnosine levels [14]. However, Tamaki and coworkers [43] were able to show an increase in carnosine with histidine in rats [43]. 

Aside from the buffering effects, carnosine has shown to have other physiological roles, including that of an effective antioxidant against oxidative stress [44]. Reactive oxygen species (ROS) can arise from exercise in several proposed mechanisms including: an increase flow of electrons in the electron transport system from increased respiration [45] or a decrease in pH can lead to oxygen being released from hemoglobin and a subsequent increase in pO_2_ in the tissues [46]. Some believe the development of ROS to be related to muscle fatigue during activity [47,48]. 

Carnosine is also linked to enzyme regulation related to activation of myosin ATPase, which is used to help maintain ATP stores [49]. Finally, carnosine has been noted to have a role in electron-contraction (E-C) coupling in skeletal muscle. An early study by Lamont and Miller [50] showed 15 mM of carnosine resulted in a significant increase in Ca^2+^ sensitivity in muscle fibers of Rana temporaria [50]. More recently, Dutka and Lamb [51] examined if carnosine affects E-C coupling in functional fibers under physiological conditions. They used mechanically skinned rat extensor digitorum longus muscle fibers. Their results showed that carnosine did not affect Ca^2+^ release from the sarcoplasmic reticulum; however, carnosine was able to increase the Ca^2+^ sensitivity of the contractile components of the muscle fibers. Authors suggested the assistance in Ca^2+^ sensitivity could help maintain force production in the later stages of fatigue once Ca^2+^ release begins to decrease. Therefore, higher levels of carnosine can help offset the decrease in Ca^2+^ as well as the accumulation of H^+^ ions during high-intensity exercise [51].

Since carnosine has a number of physiological roles, there are a number of future research opportunities. Specifically, the exact mechanism of carnosine in its role to improve exercise performance and/or reduce muscular fatigue needs to be studied. It will also be important to examine how different nutritional strategies to increase carnosine levels in the muscle may optimize physiological activity and/or exercise capacity. 

## 3. Beta-Alanine

β-ALA is a naturally occurring amino acid that is one of the precursors to carnosine, along with L-histidine. Carnosine synthetase is the enzyme used to synthesize carnosine from β-ALA and L-histidine. β-ALA is also likely to be the rate-limiting step in the synthesis of carnosine [14,52,53]. Carnosinase is the enzyme present in cells and serum that breaks down carnosine into β-ALA and L-histidine [54].

β-ALA supplementation in doses greater than 10 mg/kg of body weight has shown to cause a short period of paraesthesia with increasing severity as the dose increases. However, when a large dose around 40 mg/kg of body weight is ingested with CBEX, the paraesthesia did not occur. It is hypothesized that this side effect is a result of the rapid high peak blood plasma concentrations of β-ALA with supplementation alone, since it is not experienced when β-ALA is ingested through the diet with histidine containing dipeptides such as carnosine in meat products [7].

### 3.1. Beta-Alanine and Carnosine

As previously mentioned, β-ALA supplementation has recently been shown to significantly increase intramuscular carnosine levels, which then corresponds to improvements in exercise performance [8]. Harris and colleagues [7] examined the effects of β-ALA supplementation on human skeletal muscle carnosine concentration in a series of studies. In one study, investigators examined the effects of four weeks of β-ALA or carnosine supplementation on muscle carnosine concentrations. The supplementation protocol included consuming 800 mg of β-ALA four times a day (i.e., 2.3 g/day) for a total intake of approximately 90 g over the four week period (group I) or increasing doses of β-ALA through the supplementation period (average 6.4 g/day) for a total intake of about 146 g over the four week period (group II). The carnosine supplementation group involved consuming increasing doses of L-carnosine through the supplementation period for a total intake of 364 g of L-carnosine over the four week period which corresponded to an intake of about 143 g of β-ALA. A final group supplemented with maltodextrin as a placebo in the same frequency as the β-ALA and L-carnosine supplementation groups. A muscle biopsy was taken before and after supplementation. Results revealed that each supplement group showed significant increases in carnosine content. Mean carnosine content increase (measured in mmol·kg^-1^dm) was greatest with L-carnosine and was followed by groups II and I of β-ALA with values of 16.37 ± 3.03 (p < 0.05), 11.04 ± 2.68 (p < 0.05) and 7.80 ± 0.36 (p < 0.05) mmol∙kg^-1^dm, respectively. There was no change in the placebo group (1.87 ± 1.73, p>0.05 mmol∙kg^-1^dm). This corresponded to percent changes of 66%, 64%, 42%, and 10% for L-carnosine, group II, group I and placebo group, respectively. They also indirectly calculated the contribution of carnosine to buffering capacity between pH levels of 7.1 and 6.5 using the Henderson-Hasselbach equation. They found that after four weeks of supplementation, carnosine accounted for about 14.2%, 14.3%, and 12.6% of the total muscle buffering capacity in the L-carnosine group, group II, and group I, respectively [7].

Studies have also suggested that there does not appear to be an upper limit on increasing muscle carnosine concentrations. For example, Derave and colleagues [5] supplemented trained male sprinters with β-ALA or placebo (maltodextrin) for four to five weeks. The supplementation protocol included six daily doses of 400 mg capsules of either β-ALA or maltodextrin totaling 2.4 g/day for the first four days, 3.6 g/day for the next four days, and 4.8 g/day for the duration of the study. Interestingly, muscle carnosine levels were increased even in individuals with high resting muscle carnosine concentrations [5]. 

While β-ALA and carnosine supplementation have been reported to increase muscle carnosine levels, less is known about the time course of carnosine degradation. Carnosinase is responsible for the hydrolyzation of carnosine and is mainly present in human plasma, which is why carnosine levels are much lower in the blood than in skeletal muscle, where this enzyme is not present [26]. β-ALA supplementation in doses of 4-6 g/day over time has been shown to increase carnosine by 20-30% after two weeks, by 40-60% after four weeks, and up to 80% by ten weeks [6,55]. A study by Baguet and colleagues [56] sought to determine the loading phase of carnosine and the time course of removal. They included 20 males who supplemented with either β-ALA or maltodextrin as a placebo for five to six weeks. The investigators provided doses of 2.4 g/day for days one and two, 3.6 g/day for days three and four, and 4.8 g/day for the remainder of the study duration. Using a proton magnetic resonance spectroscopy (MRS), they measured the carnosine content in three different muscles (soleus, tibialis anterior, and gastrocnemius) at four time points (pre-supplementation, during the last week of supplementation and at weeks three and nine following the cessation of supplementation). They determined that carnosine elimination occurs relatively slowly and in a linear pattern at an average rate of 0.21 mM/week in both type I and II fibers. Authors suggest the slow clearance of carnosine is indicative of the high stability of the metabolite [56]. Table 1 provides a summary of recent studies examining the effects of β-ALA supplementation on carnosine concentrations. 

**Table 1:** Summary of the effects of β-ALA supplementation on muscle carnosine concentrations.

Authors	Population	Supplementation Protocol	Muscle Carnosine Concentration Effects	Performance Results
Baguet *et al.*, 2009 [56]	20 physically active males	5-6 weeks of β-ALA or placebo (maltodextrin)	In soleus, carnosine increased 30% (p=0.003) with β-ALA and remained stable with placebo (p=0.867)	None measured
		2.4 g/day - first 2 days	In tibialis anterior, carnosine increased 27% (p=0.005) with β-ALA and decreased 17% (p=0.05) with placebo	
		3.6 g/day - days 3-4	In gastrocnemius, carnosine increased 23% (p=0.038) and did not change with placebo (p=0.740).	
		4.8 g/day to end of study	Carnosine elimination was measured at 3 and 9 weeks after supplementation period	
			At 3 weeks, only 26.1% (in the soleus), 20.1% (in tibialis anterior) and 44.7% (in the gastrocnemius) of the increase had disappeared. There was no difference between β-ALA and placebo at this point (p=0.431)	
			At 9 weeks, carnosine levels in all 3 muscles returned to initial values	
Harris *et al.*, 2006 [7]	Study 3:21 physically active males Ages 26.1 ± 5.6 yrs	4 weeks, 4 groups (I - IV): I) 800mg β-ALA x 4 daily (avg. 3.2g daily and 89.6g 4wk total)II) 8 daily doses of either 400 or 800mg β-ALA (avg. 6.4g daily and 145.6g 4wk total)III) 8 daily doses of 1000 or 2000 mg L-carnosine (364g 4wk total L-carnosine, corresponding to 143.3g β-ALA) IV) Placebo of maltodextrin at doses to match groups II and III	Increase in carnosine concentration greatest with carnosine supplementation, followed by group II, then group II β-ALA protocols.	None measured
			Mean increase over 4 weeks (mmol·kg^-1^dm)	
			I) 7.80 ± .36 (p < .05)	
			II) 11.04 ± 2.68 (p < .05)	
			III) 16.37 ± 3.03 (p < .05)	
			IV) 1.87 ± 1.73 (p>.05)	
Derave *et al.*, 2007 [5]	15 male track athletes (sprinters) 18-24 yrs	4-5 weeks β-ALA or placebo (maltodextrin)	Soleus:	No difference between groups for 400m running performance
		2.4g/day - first 4 days	↑ 47% with β-ALA	
		3.6g/day - days 5-8	No change with placebo	
		4.8g/day to end of study	Gastrocnemius:	
			↑ 37% with β-ALA	
			No change with placebo	
Hill *et al.*, 2007 [6]	25 physically active males	10 weeks β-ALA:	β-ALA group, ↑ from 19.0 to 30.1 mmol/kg (58.8%) at 4 weeks and up to 34.7 mmol/kg (80.1%) at 10 weeks	No effect on body mass
		4g/day - wk 1	Not significant between weeks 4 and 10	↑cycling capacity time at 110% with β-ALA
		4.8g/day - wk 2		
		5.6g/day - wk 3		
		6.4g/day - wk 4-10		

### 3.2. Beta-Alanine and Exercise Performance

Increases in muscle carnosine due to β-ALA supplementation have resulted in significant effects on several variables related to exercise performance. Some of these include improved time to fatigue on a maximal cycle test [6], increased ability to sustain power output in the final ten seconds of the Wingate test [31], delayed onset of neuromuscular fatigue during incremental cycle ergometry tests as noted by increased physical working capacity (PWC_FT_), increased ventilatory threshold (VT) and time to exhaustion (TTE) [8], and improvements in muscle torque during repeated bouts of intense dynamic contractions [5].

Since studies have reported that muscle carnosine levels are typically higher in fast-twitch muscle fibers, which are most predominantly used in high-intensity anaerobic exercise bouts, it has been hypothesized that β-ALA supplementation could aid in anaerobic performance. In 2002, Suzuki and colleagues [31] performed a study that did not involve any nutritional supplementation, but simply analyzed muscle biopsy samples from the vastus lateralis before and after a 30-second maximal cycle sprint Wingate test. The muscle samples were analyzed for carnosine content. Analysis showed a direct relationship between carnosine concentration in skeletal muscle and performance on the 30-second Wingate exercise test. This relationship lends itself to the question of efficacy of β-ALA supplementation in further improving anaerobic exercise performance. 

Hill and coworkers [6] examined the effects of four and ten weeks of β-ALA supplementation on muscle carnosine concentration and high-intensity cycling capacity. They also sought to discover whether the effects were muscle type specific. Physically active males supplemented with either β-ALA or maltodextrin as a placebo. β-ALA was given in eight doses per day with increasing dose amounts during the first four weeks ranging from 250-750 mg per dose. Subjects underwent muscle biopsies and maximal cycle performance tests at various points during the study. The group supplementing with β-ALA had significantly greater muscle carnosine concentrations at four and ten weeks from 19.9 ± 1.9 to 30.1 ± 2.3 (30.4%) and 34.7 ± 3.7 (35.1%) mmol∙kg^-1^dm. There was no significant change with placebo. The change between four and ten weeks with β-ALA was not significant despite the small increase (p~0.07). The results also indicated no difference between fiber types, in that each showed similar increases in carnosine as measured by HPLC with fluorescence detection. The authors suggested that the possible benefits from β-ALA supplementation may be limited to four weeks, which is in agreement with previous findings by Suzuki and coworkers [31] who showed an increase in the ability to sustain power output after four weeks of supplementation with no additional benefits observed at ten weeks [31]. 

Limited research has examined the effects of β-ALA on sport-specific anaerobic performances. Derave and colleagues [5] studied the effects of a four week supplementation period on athletic performance, using a 400 m running race time trial. The researchers found no significant differences in performance after supplementation, but suggested this may have been due to the short time period of supplementation since it takes several weeks to induce carnosine loading. Using a proton MRS to detect muscle carnosine concentrations, investigators showed an increase in carnosine concentrations of 47% in the soleus muscle after β-ALA supplementation with no significant increase after placebo supplementation (8%). Both groups showed significant increases in carnosine concentrations in the gastrocnemius but subjects supplementing their diet with β-ALA observed a greater increase (37% versus 16%) [5]. This is in contrast to the previously discussed study that reported performance improvements after four weeks of supplementation [6]. The researchers suggested that this may be due to the possibility that in trained athletes, a 400 m running performance is not necessarily limited by the intracellular pH decrease, and therefore the buffering capabilities of the increased carnosine concentrations would not be as critical of a component [5]. 

Another recent study sought to determine whether β-ALA supplementation would affect endurance cycling performance. Van Thienen and colleagues [57] evaluated whether β-ALA supplementation would enhance the final sprint performance during endurance cycling since many competitions are won in the final seconds of the race after an all-out sprint. They studied 21 trained males who supplemented their diet with either β-ALA or a maltodextrin placebo for eight weeks. The dose gradually increased from 2 g/day for the first two weeks, 3 g/day for weeks three and four, to 4 g/day for weeks five to eight. The exercise test involved 110 minutes of cycling in ten minute stages with increasing intensity between 50-90%. Following this, the subjects performed a 30 second all-out sprint. The researchers reported that β-ALA supplementation increased sprint peak power after a two hour endurance exercise bout by 11-15% (p=0.0001) and mean power output by 5-8% (p = 0.005) [57].

In contrast to trained individuals, Smith and colleagues [58] recently examined the combined effects of six weeks of β-ALA supplementation and high-intensity interval training on endurance performance in recreationally active males. In this study, 46 participants were randomly assigned to either β-ALA or placebo supplementation groups. Both groups trained at 90-110% of their peak oxygen utilization (VO_2_peak) for the first three weeks, followed by three weeks of training at 115% VO_2_peak. During the training, they continually supplemented with 6 g/day of β-ALA or a dextrose placebo for the first three weeks and 3 g/day for the second three weeks. They showed increases in both groups for VO_2_peak, time to reach VO_2_peak, and total work done. However, the group ingesting β-ALA observed a greater increase in VO_2_peak and time to reach VO_2_peak during the second three weeks of the training protocol (p < 0.05), with no change in the placebo group. They also noted a significant increase in lean body mass for the β-ALA group after the first three weeks. These results suggest that β-ALA supplementation may enhance the effects of high-intensity interval training and improve endurance performance in untrained individuals. Additionally, Smith and colleagues [59] examined the effects of the same high-intensity interval training and β-ALA supplementation protocol described above on neuromuscular fatigue and function. The researchers reported that three weeks of the interval training was sufficient to result in a significant increase in the EMG fatigue threshold (EMG_FT_). However, β-ALA supplementation did not promote greater benefits [59]. Table 2 presents a summary of recent studies examining the effect of β-ALA supplementation and carnosine loading on exercise performance. 

**Table 2:** Summary of recent β-ALA supplementation and exercise performance studies.

Authors	Population	Supplementation Protocol	Exercise Testing Protocol	Performance Results
Baguet *et al.*, 2009 [60]	14 physically active males	4 weeks of β-ALA or placebo (maltodextrin)	Maximal ramp exercise test on cycle ergometer to determine VO_2_peak, VT and gas exchange threshold	Exercise-induced acidosis was 19% lower with β-ALA
		2.4 g/day - first 2 days	Pre and Post supplementation: 3 x 6min cycle exercise bouts at 50% ∆ power output	No difference in VO_2_ throughout exercise before or after supplementation in either group
		3.6 g/day - days 3-4		Time delay in the fast component was significantly shorter with β-ALA than placebo
		4.8 g/day to end of study		Does not support a role for acidosis in O_2_ deficit or the slow component of VO_2_ kinetics
Stout *et al.*, 2006 [4]	51 males	4 groups:	PWCM_FT_ test with EMG measurements on a cycle ergometer	β-ALA may delay the onset of neuromuscular fatigue, but no additive effects of creatine
		Placebo - 34 g dextrose		Significant increase in PWCFT with β-ALA (14.5%) and creatine plus β-ALA (11%) compared to placebo
		Creatine - 5.25 g creatine monohydrate and 34 g dextrose		
		β-ALA - 1.6 g β-ALA plus 34 g dextrose		
		β-ALA+Creatine - 5.35 g creatine monohydrate, 1.6 g β-ALA and 34 g dextrose		
		28 days of supplementation:		
		4 doses/day - days 1-6		
		2 doses/day - days 7-28		
Stout *et al.*, 2007 [8]	22 females	4 weeks β-ALA or placebo	Continuous graded exercise test on cycle ergometer for VO_2max_, ventilatory threshold , PWC_FT_ and TTE	β-ALA delays onset of NMF during incremental cycle ergometry (↑ PWC_FT_, ↑VT, ↑TTE)
	Ages:	4 divided doses/day for totals of:		
	28.9 ± 8.1 yrs (β-ALA)	3.2g/day - days 1-7		
	25.8 ± 4.0 yrs (placebo)	6.4g/day - days 8-28		
Stout *et al.*, 2008 [61]	26 elderly males and females	90 days supplementation with β-ALA or placebo (microcrystalline cellulose) 3 doses/day of: 2.4 g β-ALA or 2.4 g placebo	Continuous graded exercise test on cycle ergometer for PWC_FT_ with EMG measurements	28,5% increase in PWC_FT_ after 90 days of β-ALA
Sweeney *et al.*, 2009 [62]	19 physically active college-aged males	5 weeks β-ALA or placebo (rice flour)	2 sets of 5x5-sec sprints with 45- sec recovery between sprints and 2 min between sets performed on non-motorized treadmill at 15% body weight as resistance	No between group difference for peak or mean horizontal power
		4g/day - week 1		No difference in % fatigue
		6g/day - weeks 2-5		No difference in blood lactate pre- and post-testing between groups
Van Thienen *et al.*, 2009 [57]	17 healthy young males	8 weeks β-ALA or placebo (maltodextrin)	Simulated road race of 110 minutes intermittent endurance with intensity between 50% and 90% of the maximal lactate steady state (MLSS) in 10 minute stages. Immediately after this, they started a 10 minute time trial at 100% MLSS with voluntary increase of intensity at each minute.	β-ALA enhanced sprint power output at the end of the endurance race compared to placebo
		2 g/day - weeks 1-2		
		3 g/day - weeks 3-4		
		4 g/day - weeks 5-8		
Zoeller *et al.*, 2007 [10]	55 males ages 24.5 ± 5.3 yrs	4 weeks, 4 groups (4 doses/day for first 6 days, then 2 doses/day	Continuous graded exercise test on cycle ergometer	↑ in 5 cardio-respiratory endurance variables with creatine + β-ALA
		Placebo - 34g dextrose		Combined supplementation may delay the onset of VT and lactate threshold during incremental cycle exercise
		Creatine - 5.25g creatine monohydrate and 34g dextrose		
		β-ALA - 1.6g β-alanine and 34g dextrose		
		β-ALA plus Creatine - 5.25g creatine monohydrate, 1.6g β-ALA and 34g dextrose		

### 3.3. Beta-Alanine and Exercise Training

Many athletes incorporate resistance exercise as part of their training. Resistance-exercise has been reported to lower pH levels to around 6.8 during an exercise session [63,64]. Thus, β-ALA supplementation may provide ergogenic value to athletes engaged in resistance training due to the heavy reliance on glycolytic systems in the exercises [9]. Several recent studies have examined this hypothesis. For example, Kendrick and coworkers [9] examined the effects of ten weeks of resistance training with and without β-ALA supplementation on muscle carnosine concentration and performance measures. Subjects consumed 6.4 g/day of β-ALA or a maltodextrin placebo for ten weeks. Results revealed that β-ALA supplementation increased muscle carnosine levels by 12.8 ± 8 mmol/kg dry muscle weight in with previous research [6,7]. However, the researchers reported that β-ALA supplementation had no effects on whole body strength, isokinetic force production, muscular endurance, or body composition [9]. 

In a follow-up study, Kendrick and colleagues [65] performed a study examining the effects of four weeks of β-ALA supplementation on isokinetic training adaptations and muscle carnosine content in type I and II fibers. Fourteen male subjects were divided into two supplementation groups. Subjects ingested 800 mg of β-ALA or a maltodextrin placebo eight times per day for four weeks (6.4 g/day). Subjects trained three times a week for the first two weeks and four times a week for weeks three and four. Each session consisted of ten sets of ten maximal 90° knee extension and flexion contractions at 180°/sec on the right leg using a Kin-Com isokinetic dynamometer with one minute of rest between sets. The left leg acted as the untrained control. Muscle biopsies were obtained from the trained and untrained legs prior to and following the training and supplementation period. Results revealed that carnosine content was increased in the trained (9.6 ± 3.9 mmol/kg dry muscle) and untrained legs (6.6 ± 2.4 mmol/kg dry muscle) with no significant differences observed between groups. In addition, no significant differences were observed between carnosine concentrations in type I and type II fiber types. The researchers concluded that four weeks of isokinetic training is not effective in increasing carnosine content and that β-ALA supplementation serves to increase muscle carnosine concentration in both untrained and trained type I and type II muscle fibers [65]. Other recent studies support contentions that β-ALA supplementation can enhance training adaptations [12,58,59]. Table 3 provides a summary of recent studies on β-ALA supplementation and exercise training. 

**Table 3:** Summary of recent β-ALA supplementation and exercise training studies.

Authors	Population	Supplementation Protocol	Exercise Protocol	Muscle Carnosine Concentration Effects	Performance Results
Hoffman *et al.*, 2006 [12]	33 male strength power athletes	10 weeks	Resistance training program 4 days/week for 10 weeks	Not measured	↓ fatigue rate in CA
		Creatine β-ALA (CA) - 10.5g/day creatine monohydrate and 3.2g/day β-ALA			↑ ∆ lean body mass and % body fat
		Creatine (C) - 10.5g/day			No change in power measures
		Placebo (P) - 10.5g/day dextrose			↑ training volume in CA
Kendrick *et al.*, 2008 [9]	26 healthy males, 19-24 yrs	800mg x 8/day for 4 weeks of β-ALA or placebo (maltodextrin)	Resistance training 4days/wk for 10 weeks	β-ALA - 23.96*± *5.94 to 36.77*± *8.26 (*p < *0.0001)	No difference in whole body strength or isokinetic force
				Placebo - 29.17*± *9.82 to 27.29*± *9.52 (*p* > 0.05)	
Kendrick *et al.*, 2009 [65]	14 Vietnamese college aged students	4 weeks β-ALA or placebo (maltodextrin) 800mg x 8/day	Single legged isokinetic training	Carnosine ↑ in both trained and untrained legs with β-ALA	None measured.
			3 sessions - weeks 1-2	Training alone had no effect on carnosine levels	
			4 sessions - weeks 3-4		
			10 × 10 maximal 90° extension and flexion contractions at 180°/sec on Kin-Com		
Smith *et al.*, 2009 [59]	46 recreationally active young males	6g/day for 3 weeks, then 3g/day for 2^nd^ 3 weeks of β-ALA or placebo (dextrose)	High intensity interval training	Not measured	Training increased EMG_FT_, no additive effect with β-ALA
Smith *et al.*, 2009 [58]	46 recreationally active young males	6g/day for 3 weeks, then 3g/day for 2^nd^ 3 weeks of β-ALA or placebo (dextrose)	High intensity interval training	Not measured	↑ VO_2_peak and time to reach VO_2_peak with β-ALA
					↑ lean body mass with β-ALA

### 3.4. Beta-Alanine and Muscular Fatigue

There are several factors that play a role in muscular fatigue with high-intensity exercise. Some common theories include a disruption of the neuromuscular junction; a decrease in Ca^2+ ^release and uptake leading to the inability of muscles to contract; a depletion of fuel stores such as ATP; production of free radicals due to oxidative stress; and, the accumulation of metabolites such as H^+^[38]. Carnosine has been implicated to play a role in each of these proposed mechanisms of fatigue, but is most commonly researched for its effect on metabolite accumulation as a buffer. 

The previously mentioned study by Derave et al. [5] also examined the effects of β-ALA supplementation on isokinetic and isometric fatigue. The isokinetic protocol involved performing five sets of 30 maximal voluntary isokinetic knee extensions at 180°/sec with one minute of recovery between sets on the right leg. The isometric protocol was performed on the left leg and involved a maximal static voluntary contraction (MVC) at 45°. Once the MVC was determined, subjects performed isometric contractions at 45% of the MVC for as long as possible. Results indicated that carnosine loading significantly improved the latter stages of exercise (sets four and five of the isokinetic test). The researchers noted that the observed response with β-ALA supplementation had similar results as muscle creatine loading on muscle fatigue [66]. The authors also suggested the increase in carnosine attenuated fatigue by not only its buffering capacities, but also by its ability to improve myofibrilar Ca^2+^ sensitivity. 

Neuromuscular fatigue is defined as an increase in electrical activity of a working muscle over time [67,68,69]. The increase in electrical activity is observed by the increase in EMG amplitude and is indicative of the recruitment of more motor units and/or the increase in firing rate of the active motor units in order to attempt and sustain the given activity [69]. The accumulation of H^+^ ions is one possible explanation for this EMG response. Other possible explanations include depleted energy stores and impaired regulation of muscle cations [2,70]. deVries and coworkers [67] developed a protocol to assess neuromuscular fatigue threshold. It was termed the PWC_FT_ and examines the relationship between EMG amplitude and fatigue during cycle ergometry. This specifically measures the power output at the point of neuromuscular fatigue [8]. Subsequent studies have shown relationships between PWC_FT_ and VT as well [69,71]. 

Since it has been established in previous research that β-ALA supplementation has enhanced buffering capabilities during exercise by the subsequent increase in muscle carnosine content [5,6,7,9,31], it has been hypothesized that β-ALA supplementation may delay fatigue [8]. Until recently, this had only been shown in trained and untrained men [7]. Stout and coworkers [8] examined the effects of 28 days of β-ALA supplementation in women on PWCFT, VT, VO2max, and TTE during a cycle ergometry protocol. Subjects were assigned to supplement with either β-ALA or placebo (maltodextrin) in doses of 3.2 g daily for days one through seven and 6.4 g daily for days eight through 28. Subjects were tested prior to and following supplementation. Results showed that β-ALA supplementation increased PWCFT by 12.6%, VT by 13.9%, and time to exhaustion by 2.5%. 

Stout and colleagues [61] also recently examined the effects of three months of β-ALA supplementation on PWC_FT_ in elderly men and women. Participants supplemented with either 2.4 g β-ALA or placebo (microcrystalline cellulose) three times per day for the duration of the study. Results revealed that β-ALA supplementation increased physical working capacity in an elderly population by 28.5%. The researchers attributed these findings to an increase in muscle carnosine concentrations leading to an enhanced buffering capacity, although carnosine was not directly measured in this study [61]. The data related to by β-ALA and muscular fatigue show promise for improvements with supplementation, but still requires future research. 

### 3.5. Beta-Alanine and Creatine Supplementation

Approximately 95% of the total creatine found in the body is located in skeletal muscles, of which 40% is free creatine and 60% is phosphorylated creatine [72]. Creatine has several roles in the body during exercise, with one of the most important being as an energy source for high-intensity exercise bouts. Performances that require immediate energy (such as maximal sprints) utilize high energy phosphate, ATP and PCr that are stored in the muscles. The reversible reaction in which this energy is released is: PCr + ATP  creatine kinase  ATP + creatine [73]. Creatine supplementation enhances the initial stores and availability of PCr and therefore, theoretically would enhance the mechanisms of the phosphagen system used in high-intensity exercise and improve the shuttling of high-energy phosphates in the creatine phosphate shuttle that may potentially improve anaerobic and aerobic capacity [74,75].

During short-duration high-intensity exercise, ATP is rapidly consumed to provide energy for the given activity. In order to continue at the same intensity, the body must quickly resynthesize ATP from its byproducts. At maximal intensities, this is primarily achieved by anaerobic degradation of PCr and glycogen. The main function of PCr breakdown in this case is to act as an initial buffer and delay the reliance on glycogenolysis [66]. The decrease in maximal force production has been linked to PCr stores in a direct relationship [76]. Creatine supplementation in doses of 20-30 g/day have shown to increase skeletal creatine content by about 20%, where 20-30% of this is as PCr [77]. Creatine supplementation also shows to speed the PCr resynthesis within the first minute of recovery from intense muscular activity [78].

Creatine supplementation has been extensively studied and is known to have ergogenic properties in power and strength athletes, with recent studies showing supplementation resulting in increases in muscular strength, anaerobic power, and body mass [66,79,80,81]. In fact, the majority of long term training studies with creatine suggests an ergogenic effect with supplementation in a variety of populations including trained adolescents, adults and the elderly [11]. For example, Kreider and colleagues [82] examined the effects of 28 days of creatine supplementation during training for college football players. Subjects supplemented their diet with either a carbohydrate electrolyte placebo or this same supplement containing 15.75 g/day creatine monohydrate for 28 days while engaged in resistance-training and agility exercises. The researchers reported that the group supplementing with creatine had greater gains in fat free mass, bench press lifting volume, and repetitive sprint performance on a cycle ergometer compared to the placebo [82]. More recently, studies have examined the effects of supplementing the diet with creatine monohydrate and β-ALA on exercise performance and training adaptations. 

A study by Hoffman and colleagues [12] used male power athletes and supplemented their diet with creatine or a combination of β-ALA and creatine. The supplementation doses were 10.5 g daily of creatine monohydrate; 10.5 g daily of creatine monohydrate in combination with 3.2 g daily of β-ALA; or, 10.5 g daily of dextrose as a placebo. In addition to supplementation, subjects were involved in a ten week detailed resistance training program with workouts four days a week. The researchers reported significant improvements in body composition after ten weeks of the combined supplementation of β-ALA and creatine in conjunction with resistance training compared to creatine alone or placebo. Additionally, they showed the addition of β-ALA to creatine was able to reduce fatigue rates during training compared to creatine alone. These findings suggest that there may be additive effects of supplementation of creatine and β-ALA. 

Stout and coworkers [4] examined the effects of 28 days of β-ALA and creatine supplementation on neuromuscular fatigue and PWC_FT_. In the study, 51 men supplemented their diet with either 34 g of a dextrose placebo; 5.25 g of creatine with 34 g of dextrose; 1.6 g of β-ALA with 34 g of dextrose; or, 1.6 g of β-ALA with 5.25 g of creatine and 34 g of dextrose. Subjects ingested this dose four times a day for the first six days, and then only twice a day for the remainder of the study. Results revealed that PWC_FT_ increased in the β-ALA group with no additive effect of creatine. The researchers suggested that 28 days of β-ALA supplementation was able to delay neuromuscular fatigue during incremental cycling but this was independent of the inclusion of creatine [4].

A study by Zoeller and associates [10] examined the effects of four weeks of creatine and β-ALA supplementation on VO_2_peak, lactate threshold (LT), VT and TTE. This study had four supplementation groups including a placebo of 34 g dextrose; 5.25 g creatine monohydrate plus 34 g dextrose; 1.6 g β-ALA plus 34 g dextrose; and, a combination of 5.25 g creatine monohydrate and 1.6 g β-ALA plus 34 g dextrose. Subjects ingested these supplements four times a day for six days and then twice a day for the duration of the study. The combined creatine and β-ALA supplementation resulted in significant increases in five of the eight cardiorespiratory endurance variables tested (VO_2_ and power output at LT and VT, and percent VO_2 _peak at VT). Individually, results revealed improvements in power output at VT and total TTE for creatine alone group and improvements in power output at LT for β-ALA alone group. However, no significant effects were noted between groups. Therefore, it was concluded that the combination of creatine and β-ALA supplementation may potentially be beneficial in improving submaximal exercise performance when measured at the lactate and ventilatory thresholds [10]. Collectively, these findings suggest that there may be benefit of supplementing the diet with creatine and β-ALA but it is unclear whether these benefits are independent or additive in nature. 

### 3.6. Summary of Beta-Alanine Supplementation

The use of β-ALA in recent research has shown to increase muscle carnosine concentrations in as short as two weeks, with increasing levels with longer supplementation periods [6,55]. However, although there is strong support that β-ALA supplementation during training possesses ergogenic value, the specific mechanism of action and ergogenic value remains to be fully examined. Some studies show that β-ALA supplementation can improve high intensity exercise capacity, delay VT and/or neuromuscular fatigue, promoted greater gains in lean body mass during training, and increase VO_2_peak or time to exhaustion. On the other hand, other studies show limited effects of β-ALA supplementation on exercise performance. The combination of β-ALA and creatine monohydrate supplementation is still a new field of research with conflicting results. Additive effects were shown in one study for improving fatigue rates with a resistance training program as well as for increasing lean body mass [12]. Combined supplementation was also shown to improve VT and LT during incremental cycle exercise [10]. Other studies failed to show additive effects for variables such as anaerobic power [12] and PWC_FT_[4]. However, dosing patterns differed in these studies so it is difficult to draw definitive conclusions. 

## 4. Future Directions

Future research is needed to examine the effects of β-ALA supplementation on muscle carnosine concentrations as well as the physiological effects of increasing muscle carnosine. In this regard, more research should be conducted to understand the effects of β-ALA supplementation and corresponding increases in muscle carnosine concentrations on muscle buffering capacity, antioxidant properties, enzyme regulation, calcium regulation, exercise capacity, performance outcomes, and neuromuscular fatigue. An important direction for future research is the determination of an optimal dosing strategy of β-ALA in order to optimize increases in muscle carnosine concentrations, physiological adaptations, and performance. The current literature shows many variations in the amount and length of β-ALA supplementation; therefore, a standard strategy is still pending. Studies should also examine whether different types of exercise training may influence muscle carnosine to a greater degree in order to determine the most effective method of raising carnosine levels. Determining the correct combination of training and supplementation dose may be especially important in the athletic populations. It will also be important to study the long-term safety and efficacy of β-ALA supplementation. 

Further research is clearly warranted to assess the efficacy of β-ALA and other ergogenic nutrients such as creatine. Creatine loading significantly increases muscle phosphagen levels within a few days whereas it has been determined that β-ALA supplementation takes several weeks to increase muscle carnosine concentrations. Therefore, future research should examine effective dosing strategies to optimize the benefits of both supplements. It is also possible that different types of athletes may benefit from both β-ALA and creatine supplementation. Therefore, studies need to be conducted to examine the potential ergogenic value in trained athletes with supplementation. In addition, studies examining the effects on exercise recovery may be useful since β-ALA and creatine supplementation has been reported to delay fatigue. The majority of current research has focused on the effects in young men, with the exception of the studies by Stout and associates [8,61] which examined the effects in women and the elderly. Nevertheless, additional research is needed to examine whether age and/or gender may influence results. Another area that should be investigated is supplementing the diet with β-ALA may provide some therapeutic benefit for patients with various neuromuscular and/or muscle wasting diseases as has been reported with creatine supplementation. Finally, additional research should examine the possible synergistic effects of β-ALA with other nutrients. 

## 5. Conclusion

β-ALA supplementation is a relatively recent and growing area of research. It carries potential beneficial effects with high-intensity exercise including anaerobic sprints and resistance training. There is also potential for additive effects of β-ALA and creatine, along with other supplements, to further enhance the possible ergogenic effects. There are many possibilities for future research opportunities regarding the use of this supplement. The future of β-ALA may potentially open the door to further improvements in high-intensity exercise and sport performance in a wide range of individuals. 
